# Far-Infrared and Raman Spectroscopy Investigation of Phonon Modes in Amorphous and Crystalline Epitaxial GeTe-Sb_2_Te_3_ Alloys

**DOI:** 10.1038/srep28560

**Published:** 2016-06-24

**Authors:** V. Bragaglia, K. Holldack, J. E. Boschker, F. Arciprete, E. Zallo, T. Flissikowski, R. Calarco

**Affiliations:** 1 Paul-Drude-Institut für Festkörperelektronik, Hausvogteiplatz 5-7, 10117 Berlin, Germany; 2 Helmholtz-Zentrum Berlin für Materialien und Energie GmbH, Albert-Einstein-Str. 15, D-12489, Berlin, Germany; 3 Dipartimento di Fisica, Università di Roma “Tor Vergata”, Via della Ricerca Scientifica 1, I-00133 Rome, Italy

## Abstract

A combination of far-infrared and Raman spectroscopy is employed to investigate vibrational modes and the carrier behavior in amorphous and crystalline ordered GeTe-Sb_2_Te_3_ alloys (GST) epitaxially grown on Si(111). The infrared active GST mode is not observed in the Raman spectra and vice versa, indication of the fact that inversion symmetry is preserved in the metastable cubic phase in accordance with the F
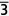
m3 space group. For the trigonal phase, instead, a partial symmetry break due to Ge/Sb mixed anion layers is observed. By studying the crystallization process upon annealing with both the techniques, we identify temperature regions corresponding to the occurrence of different phases as well as the transition from one phase to the next. Activation energies of 0.43 eV and 0.08 eV for the electron conduction are obtained for both cubic and trigonal phases, respectively. In addition a metal-insulator transition is clearly identified to occur at the onset of the transition between the disordered and the ordered cubic phase.

GeTe-Sb_2_Te_3_ alloys (GST) are highly interesting compounds in terms of both fundamental investigations on phase change properties and technological applications in memory devices^1^,^2^. In phase change memories devices, the metastable crystalline phase of GST is utilized as the memory SET state, whereas the RESET state is realized in the amorphous phase^1^. The dramatic differences in physical properties such as resistivity between the amorphous and crystalline phase of GST are primarily due to the change in the character of the chemical bonds from covalent in the amorphous to resonant in the crystalline phase^3^. In addition to the amorphous to crystalline transition, a metal-insulator-transition (MIT) is evidenced^4^,^5^,^6^ within the metastable phase of GST (disordered cubic to ordered cubic). Both changes in bonding as well as in symmetry are perfectly suited to be investigated by Raman scattering as well as for far-infrared (FIR) spectroscopy. In addition, the included THz spectral range is particularly sensitive to conductivity changes depending on the phase. However, while Raman spectroscopy was often employed for such alloys^7^,^8^,^9^,^10^,^11^, the latter technique was mostly applied to Sb_2_Te_3_^10^, with the exception of dynamic experiments^12^.

Recently, we were able to achieve a fundamental advance in the fabrication of GST by molecular beam epitaxy (MBE) resulting in as-deposited single-crystalline material with out-of-plane stacking of vacancy layers^6^, a highly-ordered structure with both cubic and rhombohedral stacking. Most interestingly, highly-ordered cubic GST (c-GST) is also obtained-through annealing treatment of amorphous GST (a-GST) deposited on a crystalline substrate^6^,^13^. In this case, it is possible to slowly tune the structural transitions (amorphous to crystalline and cubic to trigonal) to identify both the change in bonding and symmetry.

In this study we performed a combination of FIR and Raman spectroscopy to investigate vibrational modes in amorphous and crystalline epitaxially grown as well as annealed GST samples. We assign the observed phonon modes to the different crystalline phases by comparing temperature dependent FIR and Raman spectra. By FIR absorption we discriminate between the contributions of phonons and free carrier delocalization upon sample annealing for both amorphous to crystalline and insulating to metal transitions.

## Results and Discussion

**Figure 1:**
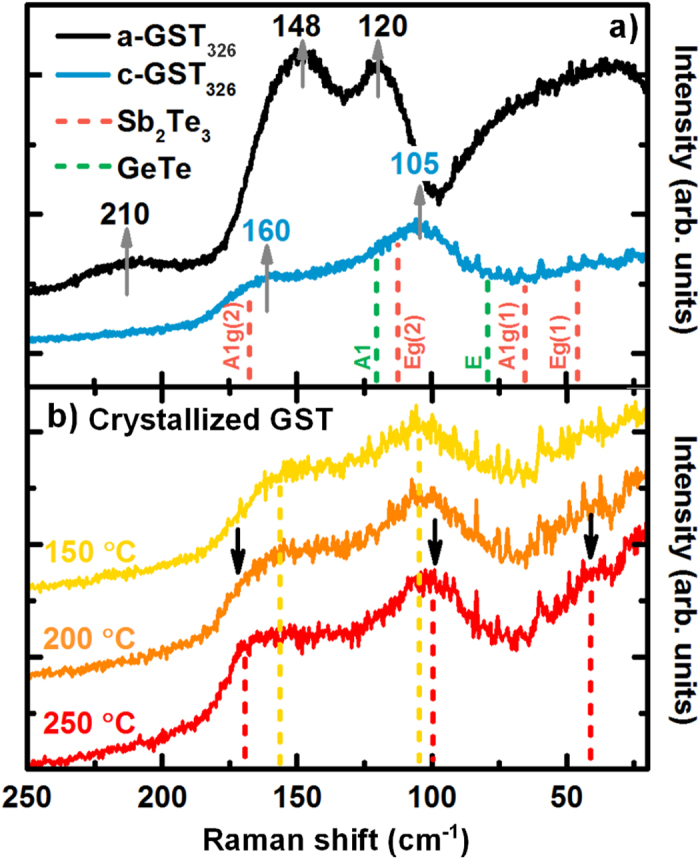
(**a**) Comparison of Raman spectra for a-GST326 (black) and as grown c-GST326 (blue); gray arrows highlight the mode positions. Sb_2_Te_3_ (red) and GeTe (green) Raman mode positions are plotted as references. (**b**) Raman spectra of crystallizing a-GST326 for three different temperatures. Modes of metastable c-GST326 are highlighted with yellow dashed lines. Upon increasing the temperature new modes appear, indication of the transition from c- to t-GST. At T = 200 °C the characteristic mode of t-GST (~170 cm^−1^) appears (red dashed lines). In the red curve (T = 200 °C) the two arrows highlight the other two modes of the t-GST.

In Fig. 1(a) Raman spectra of a- and c-GST326 samples are presented in the spectral range from 30 cm^−1^ to 250 cm^−1^. The a-GST spectrum presents the characteristic Bose peak (30–100 cm^−1^)^9^, and two modes centered at 120 and 148 cm^−1^, assigned to vibrations of defective octahedra^14^. The broad feature at 210 cm^−1^is ascribed to vibrations of tetrahedra^14^. In the c-GST spectrum two strong broad modes centered at 105 and 160 cm^−1^ are present. Polarization dependent measurements (not shown) help to assign the modes to E_g_ (105 cm^−1^) and A_1g_ (160 cm^−1^). Such modes are characteristic of the metastable cubic c-GST phase (point group m
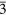
m) in accordance with previous studies^14^. According to the F
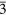
m3 space group expected for metastable c-GST, and the sites occupancy given from Nonaka *et al*.^15^, no Raman active modes should be allowed, and only the T_1u_ (IR active mode, see later in the text) is expected. The fact that such vibrations (E_g_ and A_1g_) are observed and are broad, is attributed to the presence of vacancies and defects that are responsible for the local symmetry breaking^14^. The fluctuation of compositions has been evidenced in a formerly published paper^6^ and is intrinsic for certain GST compositions. Due to the broad nature of c-GST vibrational modes, the treatment of such fluctuation is not obvious. Both binary compounds constituting GST are measured for reference purposes and the peak position are displayed in Fig. 1(a) with green (α-GeTe - R3m space group) and red (Sb_2_Te_3_–R
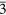
m space group) dotted lines. The Raman modes of the metastable c-GST326 [Fig. 1(a) blue curve] are prevalently arising from the Sb_2_Te_3_ modes A_1g_(2) and E_g_(2) slightly shifted (~7 cm^−1^) toward lower energies, while the modes of GeTe do not strongly contribute, due to their lower polarizability if compared to Sb_2_Te_3_, as already reported in literature^9^,^14^ (see Table 1 for peak positions, mode assignments and their IR and Raman activities). In particular the mode at 160 cm^−1^ has a one-mode behavior (Sb_2_Te_3_-like) while the mode at 105 cm^−1^ has a two modes behavior (Sb_2_Te_3_-like and GeTe-like). The mode position shift of the c-GST326, compared to the binary constituents, is the indication of mode wavenumbers compositional dependence, similarly as for transition metal di-chalcogenides^16^.

In order to study the temperature dependence of the vibrational modes, annealing of a-GST326 was performed *in-situ* during Raman data acquisition. A representative selection of the resulting spectra is plotted in Fig. 1(b). At T = 150 °C (yellow curve) characteristic modes of the cubic phase (dotted yellow lines) compare well with those reported in Fig. 1(a) for the as grown c-GST326. At T = 250 °C the film is transformed into the trigonal phase, t-GST (red curve), for which three modes are identified^17^: two evident at 170 cm^−1^ (A) and 100 cm^−1^ (E) and a faint one at 45 cm^−1^ (A). The mode at 170 cm^−1^ starts to be visible in the spectra at T = 200 °C (see arrow on the orange curve), and could be associated to vacancies ordering into layers which breaks locally the cubic symmetry, and will transform into van der Waals gaps once the t-GST is achieved (red curve). The progressive creation of ordered vacancy layers obscures the unequivocal assignment of a specific space group within the transition region. As reported in our previous studies^13^, we cannot exclude possible compositional rearrangement in the stable phase.

**Figure 2:**
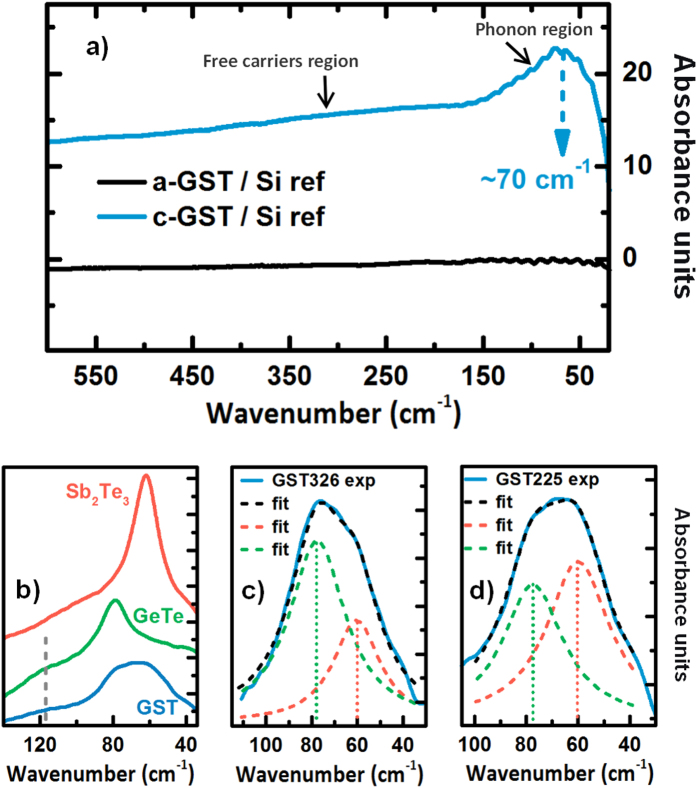
(**a**) FIR absorbance spectra for a-GST326 and c-GST326, black and blue curves, respectively. The spectra are normalized to the Si substrate. (**b**) Zoom around GST225 absorption feature (30 to 140 cm^−1^) (blue), with Sb_2_Te_3_ (red) and GeTe (green) spectra as references. (**c**) Fit of the GST326 experimental curve using two Lorentzian peaks centered at the position of the Sb_2_Te_3_ (red) and GeTe (blue) modes. (**d**) Fit of GST225 for comparison.

Figure 2(a) shows the FIR absorbance of MBE gorwn a-GST326 (black curve) and c-GST326 (blue curve) [Absorbance = −Log (T_GST_/T_Si_) where T_GST_ and T_Si_ are the GST and Si transmitted intensities]. Within the resolution of the measurement, no absorption in the whole spectral range is measured for a-GST326, in line with the absence of free carriers in the amorphous phase and due to a random distribution of local dipoles. On the contrary, c-GST326 shows a strong absorption on the whole spectral range which is an indication of metallic behavior with high free carrier concentration (~10^20^ cm^−3^ measured by low temperature transport measurement) and a broad (FWHM ~40 cm^−1^) absorption feature around 70 cm^−1^. In a-GST carriers are localized^3^,^5^,^18^ while in c-GST delocalized electrons allows for the conduction^4^,^5^. For c-GST we divide the spectral range into two main regions: the phonon dominated region between 30 and 150 cm^−1^, that we assume to be sensitive to the lattice transformations upon phase transitions, and the free carrier dominated region above 150 cm^−1^.

Figure 2(b) shows a dedicated measurement with high resolution around the phonon related absorption feature for MBE grown c-GST326 (blue), GeTe (green) and Sb_2_Te_3_ (red). Sb_2_Te_3_ displays a peak at 62 cm^−1^, which corresponds to the A_1u_ mode for the symmetry R
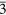
m, while α-GeTe presents two peaks at 79 cm^−1^ and 119 cm^−1^ (grey dashed line in the plot), attributed to the E and A_1u_ modes, respectively (see also Raman spectrum). In the case of c-GST326, the IR phonon mode centered at ~70 cm^−1^, according to the m
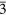
m point group of the cubic symmetry, is attributed to a T_1u_. However, as already mentioned, vacancies, defects and distortion of bonds could break the inversion symmetry predicted by the space group, leading to a mixed nature of the phonon modes^19^. The broad mode T_1u_ of c-GST326 is composed by the superposition of the phonon modes of the binary constituent compounds, E and A_1u_ for GeTe and Sb_2_Te_3_, respectively, [see Fig. 2(c,d)] since it is possible to best fit the peak with two Lorentzian functions centered at the experimental positions of the GeTe and Sb_2_Te_3_ modes, indicating a two-modes type behavior (Sb_2_Te_3_ and GeTe-like). As opposed to Raman spectroscopy, the two binary component modes show no wavenumber dependency on GST composition. The latter is accounted only in the relative intensities of the two modes. In particular the main contribution, comparing peak intensities, is the E mode of GeTe, for c-GST326 [see Fig. 2(c)] where more Ge-Te than Sb-Te bonds are expected. Instead, for the c-GST225 case [see Fig. 2(d)] a slightly higher intensity of the Sb_2_Te_3_ component is visible. FIR spectroscopy thus helps in the quantification of compositional changes.

**Figure 3:**
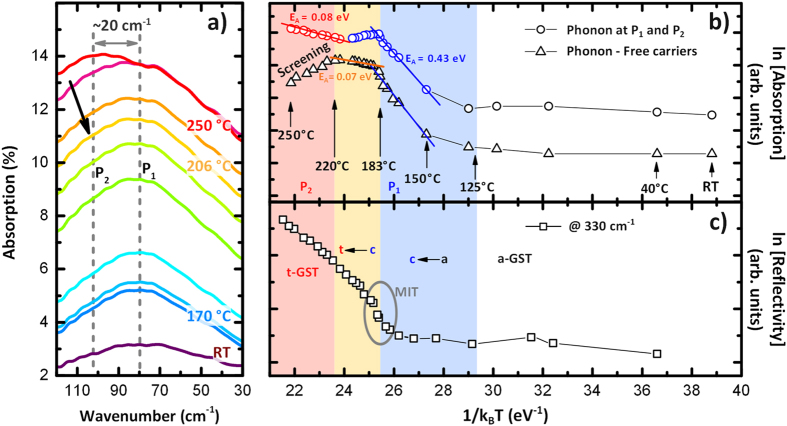
(**a**) Temperature dependent absorption spectra of crystallizing a-GST326 around the main absorption feature. (**b**) Arrhenius plot based on the intensity evolution of the phonon dominated region: peak P_1_ for c-GST326 and P_2_ for t-GST (empty circles), and difference between phonon and carrier dominated region (at 330 cm^−1^) intensities evolution (empty triangles). Activation energy of the conduction process (blue for cubic- and red for t-GST) are obtained after fitting. (**c**) Arrhenius plot of the evolution of the reflectivity at 330 cm^−1^. Four main regions are visible in (**b,c**): white for a-GST326, blue for c-GST326, orange for the transition from c- to t-GST and red for t-GST.

Crystallization by annealing of a-GST326 was also studied by *in-situ* temperature dependent FIR spectroscopy. The phonon dominated region is shown in Fig. 3(a) where the absorption (Absorption = 1 − (T_GST_ − T_Si_)/T_0_, with T_0_ the incident intensity) increases with the annealing temperature from a value close to zero till a maximum value of 14%. Please note that at high resolution, a faint mode at 80 cm^−1^ is visible in a-GST326, indicative of the presence of short range ordering. In addition, starting at 206 °C the main peak at 80 cm^−1^ (P_1_, see grey dotted line) attributed to c-GST326 decreases while a new mode at ~100 cm^−1^ ascribed to t-GST emerges (P_2_ black arrow and grey dotted line in Fig. 3(a)). P_2_ becomes more evident at higher annealing temperature [red curve Fig. 3(a)] when the film is completely trigonal (T > 225 °C), in accordance with Raman spectra in Fig. 1(b).

As-grown c-GST326 as well as a-GST326 crystallized at low annealing temperatures [T = 150 °C in Fig. 1(b)] belong to the m
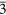
m point group with the exclusion (IR vs. Raman) selection rule preserved. Once the cubic to trigonal phase transition takes place, if we exclude a transition region where both modes characteristic of the two phases (P_1_ at 80 cm^−1^ for the cubic and P_2_ at 100 cm^−1^ for the trigonal) coexist and the symmetry determination is not possible, the exclusion selection rule seems not to hold and the mode at 100 cm^−1^ appears in both Raman (see Fig. 1(b)) and IR spectra (clearly evident in the completely t-GST annealed at 250 °C). According to literature, symmetry change between the two phases takes place from the m
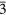
m to the expected 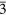
m point group, with t-GST belonging to the space group R
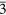
m or P
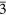
m1 depending on the composition^20^, for which mutual exclusion selection rules are valid. However, in Sosso *et al*.^17^ the effect of mixed Ge/Sb layers is shown to induce a partial break of the symmetry, from P
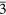
m1 to a lower symmetry state, where the Pm symmetry is preserved, allowing the double character (both Raman and IR) of the modes.

Several Arrhenius plots are extracted from the absorption spectra and plotted as a function of 1/k_B_T in Fig. 3(b,c). The contribution arising from phonons (see Fig. 3(a)) is shown by circles, blue for P_1_ (c-GST326) and red for P_2_ (t-GST). Four main regions can be identified: white for a-GST, blue for the transition region from a-GST to c-GST, orange for the transition from c- to t-GST and red for t-GST. The annealing temperature ranges agree well with that of our previous XRD studies^6^,^13^. Please note that a full disordered c-GST326 is obtained at 130 °C 13 and it is ordered at about 183 °C 6, while t-GST is present already at 225 °C. From the slope of the curves in the linear blue region an activation energy for the conduction of c-GST326 is extracted, this giving E_A_ = 0.43 eV, a value which compares well with literature^21^,^22^. In the red region (t-GST) the slope of P_2_ has an activation energy of E_A_ = 0.08 eV, indication of an enhanced metallic behavior. Additional information can be obtained if we consider the difference (triangles in Fig. 3(b)) between the absorption of phonons (P_1_ and P_2_) and free carriers (at 330 cm^−1^). For temperatures lower than 183 °C the P_1_ phonon evolution, indication of a-GST326 to c-GST326 phase transformation, is dominant, as the two curves (circles and triangles) display the same shape. Above 220 °C, the free carriers are screening the P_2_ phonon, suggesting longitudinal nature for P_2_ in the trigonal phase. In the transition region between c- and t-GST the progressive ordering of vacancies into layers, till the formation of van der Waals gaps, leads to free carrier delocalization and metallic behavior with an activation energy E_A _= 0.07  eV (orange line).

In Fig. 3(c) we display the Arrhenius plot of the reflectivity at 330 cm^−1^. Although the absolute values could be not reliable as the measured sample is very thin (~30 nm), however, we clearly see that the reflectivity increases continuously during annealing induced transition from c-GST to t-GST, indication of the enhanced metallic behavior of the sample. The reflectivity increases significantly only at about T = 176 °C that we can identify as the temperature for the MIT to occur, as it corresponds to the sudden increase in conduction^6^. Above T = 183 °C a second trend can be identified, and is ascribed to the cubic to trigonal phase transition that proceeds gradually. Furthermore, the increase of reflectivity above 220 °C reflects the increase of free carrier delocalization and confirms their dominant role in screening the phonon.

In conclusions within this study we assign the symmetries to the crystalline phases of GST326 by comparing FIR and Raman temperature dependent spectra. Ordered c-GST is ascribed to the F
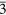
m3 space group, while as for t-GST the inversion selection rules do not hold, a partial symmetry breaking due to Ge/Sb mixed anion layers^17^ occurs. We have also demonstrated that FIR spectroscopy is sensitive to composition difference in as grown crystalline GST samples and helps in quantification of conduction enhancement/carrier behavior upon phase transitions. Furthermore, by studying the FIR absorption evolution upon annealing, we discriminate the contributions of phonons, and free carrier delocalization for the conduction of c-GST326 and t-GST, as well as for the transition regions a-GST326 to c-GST326 and c- to t-GST. In addition, from the reflectivity change the MIT is clearly identified and occurs at the onset between disordered to ordered cubic phase, in line with our previous results^6^.

## Methods

### MBE growth

A series of Sb_2_Te_3_^23^, GeTe^24^,^25^, a-GST and metastable c-GST films with compositions Ge : 3 Sb : 2 Te : 6 (326) and Ge : 2 Sb : 2 Te : 5 (225), unintentionally doped, were deposited by MBE^23^ on a highly resistive (5 kΩcm^−1^) crystalline Si(111)-(√3 × √3)R30°-Sb surface^23^ with a thickness ranging between 30 and 40 nm. The samples were capped with 30 nm of Si_3_N_4_ by sputtering to prevent oxidation of the films.

### XRD

Samples were characterized by means of *ex-situ* X-ray diffraction (XRD), utilizing a PANalytical X’ Pert PRO MRD diffractometer with Ge (220) hybrid monochromator, employing a Cu Kα_1_ radiation (λ = 1.540598 Å). XRD revealed that the crystalline GST films are quasi single crystalline^13^,^26^ with vacancies ordered into layers^6^.

### Raman measurements

Raman spectra were acquired exciting samples with the 632.8 nm line of a He-Ne laser and the scattered light was analyzed using a spectrometer equipped with an LN_2_-cooled charge-coupled device detector. The spectra were recorded in backscattering geometry in crossed and parallel polarization configurations. For the temperature dependent measurements a heating stage (THMS600 by Linkam) was employed during Raman spectra acquisition.

### FIR measurements

Measurements in the far-infrared regime were carried out under vacuum conditions both in transmission and reflection geometries using a high-resolution Fourier transform infrared spectrometer (BRUKER IFS 125HR) of the THz beamline at Helmholtz-Zentrum Berlin (BESSY II)^27^. The spectral range in the presented experiments covered wavenumbers between 30 and 650 cm^−1^ (i.e., frequencies from 0.9 to 19 THz) and was limited by the selected source (internal Hg-lamp), the 6 μm multilayer-mylar beamsplitter and the detector, a 4.2 K Si-Bolometer. A copper block heating stage was employed during *in-situ* temperature dependent FIR measurements.

## Additional Information

**How to cite this article**: Bragaglia, V. *et al*. Far-Infrared and Raman Spectroscopy Investigation of Phonon Modes in Amorphous and Crystalline Epitaxial GeTe-Sb_2_Te_3_ Alloys. *Sci. Rep*. **6**, 28560; doi: 10.1038/srep28560 (2016).

## Figures and Tables

**Table 1:** Vibrational mode assignment and position for a-GST326, c-GST (both 225 and 326), t-GST, Sb_2_Te_3_ and GeTe.

Mode	(cm^−1^)	IR	Raman
a-GST			
A1	120	no	yes
A1	148	no	yes
	210	no	yes
c-GST			
T_1u_	70	yes	no
E_g_	105	no	yes
	120	yes	no
A_1g_	160	no	yes
t-GST			
A-type	45	no	yes
E-type	100	yes	yes
A-type	170	no	yes
Sb_2_Te_3_			
E_g_(1)	48	no	yes
A_u_(1)	62	yes	no
A_1g_(1)	67	no	yes
E_g_(2)	113	no	yes
A_1g_(2)	165	no	yes
GeTe			
E	79	yes	yes
A1	119	yes	yes

IR and Raman activity are specified. Note that the position of T_1u_ for c-GST is reported as the convolution of the two peaks at 60 cm^−1^ (GST225) and 80 cm^−1^ (GST326), according to Fig. 2. See text for details.
